# When Diverticulosis Crosses Boundaries: A Case of Appendiceal Involvement in Colonic Diverticular Disease

**DOI:** 10.7759/cureus.108145

**Published:** 2026-05-02

**Authors:** Sherif Elgohary, Fawaz Shaik, Muhammad Ahmad, Hesham Aljohary, Ahmad Zarour

**Affiliations:** 1 College of Medicine, Qatar University, Hamad Medical Corporation, Doha, QAT; 2 College of Medicine, Qatar University, Doha, QAT; 3 Acute Care Surgery, Hamad Medical Corporation, Doha, QAT; 4 Surgery, Hamad Medical Corporation, Doha, QAT

**Keywords:** appendectomy, appendectomy variants, appendiceal diverticulosis, appendicular diverticulitis, appendicular diverticulosis, diverticulitis, diverticulosis, diverticulosis of the appendix, right iliac fossa pain, right-sided diverticulitis

## Abstract

Appendiceal diverticulosis is a rare entity that often mimics acute appendicitis and is typically diagnosed postoperatively. It carries a higher risk of perforation and mortality compared to simple appendicitis, making recognition clinically important.

We report a case of a 55-year-old male presenting with a 2-week history of intermittent right iliac fossa pain with acute exacerbation. Imaging demonstrated a distended, thick-walled appendix without clear signs of acute inflammation. The patient underwent a laparoscopic appendectomy for suspected chronic appendicitis. Intraoperative findings showed mild inflammatory changes without perforation. Histopathological examination revealed appendiceal diverticulosis with focal acute diverticulitis. Notably, the patient also had coexisting colonic diverticulosis. The postoperative course was uneventful.

Appendiceal diverticulosis is difficult to diagnose preoperatively due to nonspecific clinical and radiologic findings. Histopathology remains the gold standard for diagnosis. This condition is associated with a significantly increased risk of perforation and may coexist with colonic diverticular disease, suggesting a possible shared pathophysiological mechanism.

This case highlights the importance of considering appendiceal diverticulosis in atypical or chronic presentations of appendiceal pathology. Routine histopathological evaluation is essential for diagnosis and for identifying potential associations with broader diverticular disease.

## Introduction

Diverticular disease represents one of the most prevalent gastrointestinal disorders, affecting millions of patients worldwide [1,2]. While colonic diverticulosis is common, presentation in the appendix is an exceptionally rare condition, identified in approximately 0.004% to 2.1% of appendectomy specimens [3].

First described by pathologist T.H. Kelynack in 1893, appendiceal diverticulosis continues to pose significant diagnostic challenges due to its clinical presentation closely mimicking acute appendicitis [4]. Although the vermiform appendix has been described as ‘a useless and harmful organ,’ and Kelynack (1893) even referred to it as obsolete and out of date, predicting that in the future human intestine, this structure would no longer be found attached to the cecum; however, it continues to be a source of significant morbidity and mortality in humans [4].

Diverticulosis refers to the presence of sac-like outpouchings of the bowel wall, which may become inflamed, resulting in diverticulitis. In the case of the appendix, appendiceal diverticula are classified as either congenital (true) diverticula or acquired pseudodiverticula, with acquired pseudodiverticula being more common [3,5]. Appendiceal diverticulosis patients present at an older age than those with acute appendicitis, with mean ages of 38.8 compared to 19.5 years, respectively [6,7]. Evidence shows that perforation and mortality risk are elevated as compared to appendicitis alone, with some studies reporting perforation rates of 30% to 70% and a 30-fold increased mortality risk [8,9]. Despite advances in imaging modalities, preoperative diagnosis remains infrequent, with most cases being identified intraoperatively or confirmed through histopathological examination.

This article presents a rare case of appendiceal diverticulosis in a 55-year-old male patient managed at Hamad Medical Corporation, Qatar.

## Case presentation

**Figure 1:**
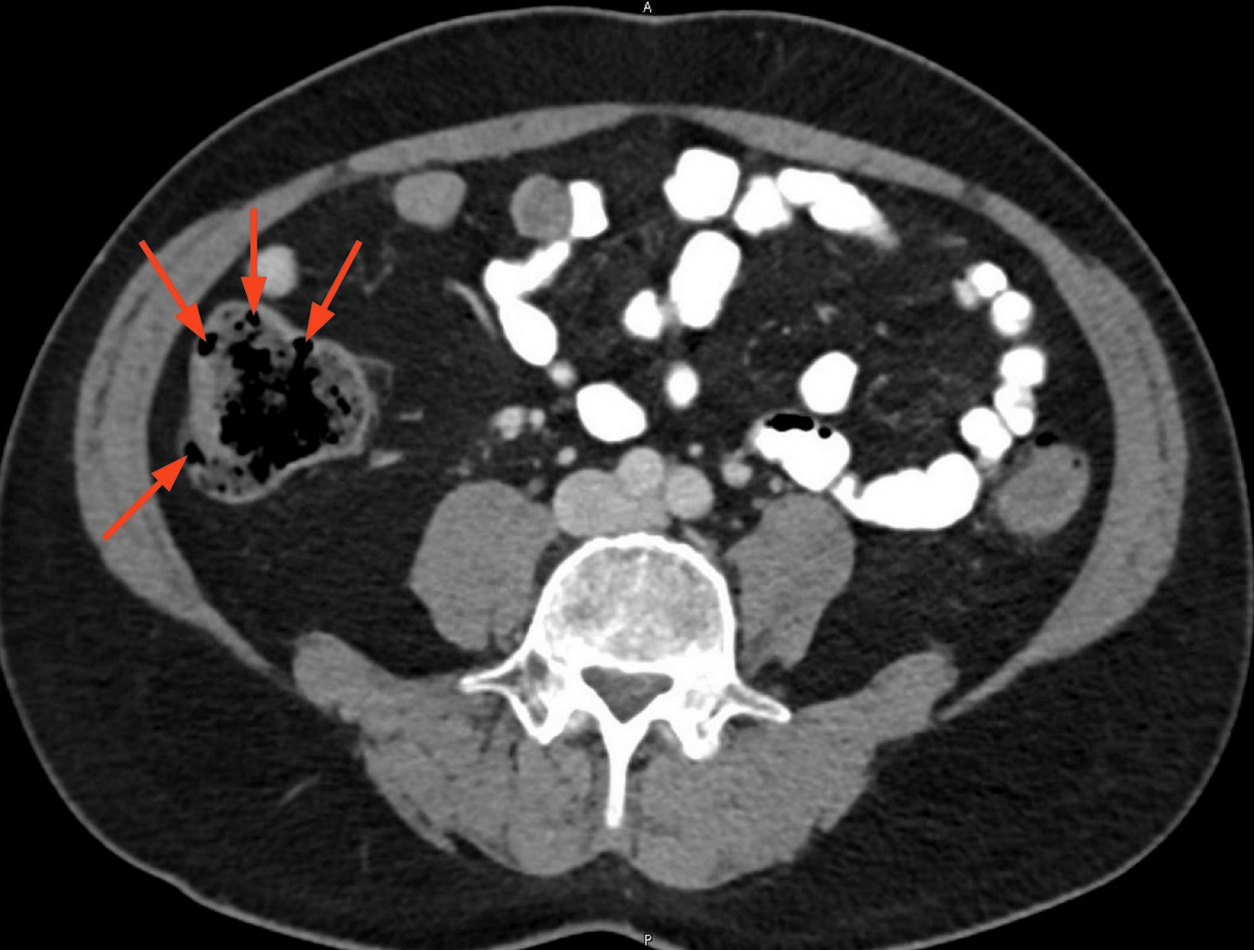
Axial CT scan of the abdomen showing cecal diverticulosis CT image demonstrating multiple cecal diverticula (red arrows), seen as air-containing outpouchings within the wall of the cecum.

A 55-year-old Jordanian male presented to the Emergency Department with a 2-week history of intermittent right lower quadrant pain associated with meals. On admission, the patient was afebrile and hemodynamically stable. Abdominal examination revealed tenderness in the right iliac fossa. A contrast-enhanced CT scan was done, which demonstrated a thick-walled appendix measuring approximately 10 mm in diameter, without evidence of any peri-appendiceal fat stranding, abscess formation, or appendicolith. In contrast, the cecum showed multiple small outpouchings along its wall, consistent with diverticulosis (Figure 1).

**Figure 2:**
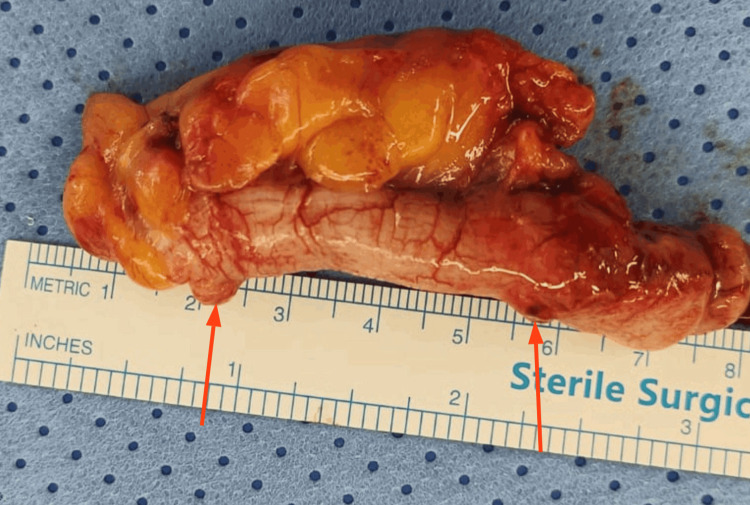
Gross resected appendix showing diverticular outpouchings (red arrows) with surrounding inflammation The specimen measures 5.0 cm in length and 1.5 cm in diameter, with a smooth, glistening tan-gray serosal surface. On sectioning, the lumen is patent and the mucosa appears unremarkable, with no obvious gross lesions.

**Figure 3:**
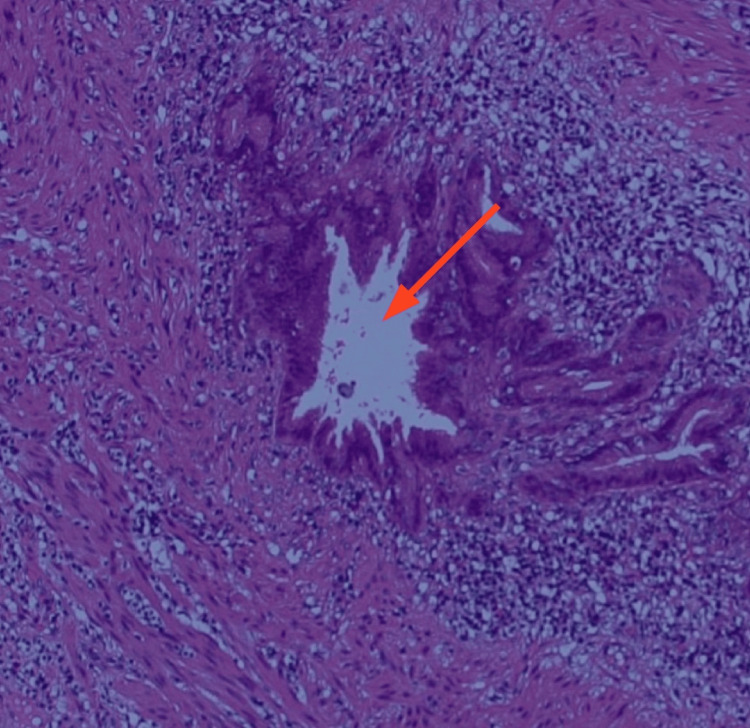
Histopathological image of appendiceal diverticulum Histologic section of the appendix stained with hematoxylin and eosin (H&E) showing an appendiceal diverticulum (red arrow), characterized by a mucosal outpouching with associated inflammatory cell infiltrate (Magnification 100x).

The patient was given intravenous fluids and a round of broad-spectrum antibiotics in preparation for an emergency laparoscopic appendectomy. Intraoperatively, a paracecal appendix with features of chronic inflammation and superimposed acute changes (American Association for the Surgery of Trauma (AAST) Grade I) was identified [10]. On macroscopic examination, the appendix appeared mildly enlarged, with a smooth, intact serosal surface and no obvious mucosal abnormalities (Figure 2). However, histopathological analysis revealed appendiceal diverticulosis with focal acute diverticulitis, a rare finding that helps explain the patient’s long-standing intermittent symptoms as well as the recent acute flare (Figure 3). Additionally, neoplasia with regard to the specimen was not investigated.

The patient recovered smoothly after surgery, with no complications, was discharged the day after the operation, and had no symptoms at their two-week follow-up visit.

## Discussion

Appendiceal diverticulosis remains a rare pathological finding, identified in only 0.004% to 2.1% of appendectomy specimens [3]. These are broadly categorized into congenital and acquired types. Congenital diverticula are "true" diverticula containing all appendiceal wall layers, while the far more common acquired pseudodiverticula consist of mucosa and submucosa herniating through defects in the muscularis propria [3,5]. These defects typically occur at vascular hiatuses along the mesenteric border where the vasa recta penetrate the muscular wall [3,6].

The patient’s age (55 years) and clinical history are highly characteristic of this pathology. While acute appendicitis is a disease of the young (mean age 19.5 years), appendiceal diverticulosis typically presents in the fourth or fifth decade of life [6,7]. The two-week history of intermittent right iliac fossa pain also mirrors the "insidious" and "episodic" nature of the disease described in the literature, which lacks the classic migratory periumbilical pain and gastrointestinal symptoms like nausea or vomiting seen in simple appendicitis [3,8].

Preoperative diagnosis is notoriously difficult. In this case, CT showed appendiceal thickening but failed to visualize the diverticula. This diagnostic gap is common; small diverticula, often measuring only 2-5 mm, can be easily masked by peri-appendiceal fat stranding or secondary inflammation [3,9]. Consequently, the diagnosis is frequently a retrospective one made by the pathologist.

Appendiceal diverticulitis has been classified into four subtypes; this case aligns with type 2 since both appendicitis and diverticula are present [8]. This subtype suggests a pathophysiological sequence where the initial inflammation or obstruction of a diverticulum leads to secondary luminal obstruction of the appendix itself [9]. Recognizing this condition is clinically important. Because pseudodiverticula lack a protective muscularis propria, they may be more susceptible to complications. Perforation rates are reported to be 4 times higher than in standard appendicitis (30%-70%) and have been associated with an increased mortality risk in the literature [8,9].

The literature consistently highlights a strong association between appendiceal diverticula and underlying neoplasms [3]. Incidental tumors, such as carcinoids, mucinous adenomas, or even adenocarcinomas, are reported in 29% to 48% of these cases [3,11]. The diverticula may act as a "proxy" for increased intraluminal pressure caused by an underlying, undetected tumor. This necessitates a meticulous histological review of the entire specimen, even when macroscopic findings appear benign, to rule out occult malignancy or precursors to pseudomyxoma peritonei [12,13].

Our case is particularly unique due to the concurrent extensive colonic diverticulosis. Traditional academic views suggest that appendiceal and colonic diverticulosis are unrelated entities with distinct pathogeneses [6,11]. However, the "crossing of boundaries" seen here suggests that in certain patients, a possible association, perhaps related to connective tissue laxity or generalized motility disorders, may lead to diverticular formation throughout the gastrointestinal tract [14].

Laparoscopic appendectomy was successfully employed here and remains the definitive treatment for both symptomatic and incidental cases discovered intraoperatively [13]. Given the reported risk of perforation and the association with underlying neoplasms, some studies have suggested consideration of appendectomy when appendiceal diverticulosis is incidentally identified, even in asymptomatic patients [3,14]. However, this recommendation remains based on limited evidence. This case reinforces the necessity of maintaining a high index of suspicion in older patients with atypical right-sided pain and underscores the indispensable role of the pathologist in the definitive management of diverticular disease.

## Conclusions

Appendiceal diverticulosis is an uncommon entity that is often identified incidentally on histopathological examination following appendectomy. It may present with clinical and radiological features indistinguishable from acute appendicitis, making preoperative diagnosis challenging. This case highlights the coexistence of appendiceal and colonic diverticular disease, which may be an incidental finding or suggest a possible association that requires further study. Given the reported association with perforation and underlying neoplasia in the literature, careful histopathological examination of appendectomy specimens remains essential for accurate diagnosis and exclusion of significant pathology.
